# Effect of combined wet alkaline mechanical pretreatment on enzymatic hydrolysis of corn stover and its mechanism

**DOI:** 10.1186/s13068-022-02130-0

**Published:** 2022-03-17

**Authors:** Jie Yang, Chongfeng Gao, Xueqi Yang, Yanfu Su, Suan Shi, Lujia Han

**Affiliations:** Engineering Laboratory for Agro Biomass Recycling & Valorizing, College of Engineering, China Agricultural University, Box 191, Beijing, 100083 China

**Keywords:** Corn stover, Mechanical-chemistry treatment, NaOH pretreatment, Ball milling, Enzymatic hydrolysis, Correlation analysis

## Abstract

**Background:**

To further optimize the mechanochemical pretreatment process, a combined wet alkaline mechanical pretreatment of corn stover was proposed with a short time and less chemical consumption at room temperature.

**Results:**

The combined alkaline mechanical pretreatment significantly enhanced enzymatic hydrolysis resulting a highest glucose yield (*Y*_G_) of 91.9% with 3% NaOH and ball milling (BM) for 10 min. At this optimal condition, 44.4% lignin was removed and major portion of cellulose was retained (86.6%). The prehydrolysate contained by-products such as monosaccharides, oligosaccharides, acetic acid, and lignin but no furfural and 5-HMF. The alkaline concentration showed a significant impact on glucose yield, while the BM time was less important. Quantitative correlation analysis showed that *Y*_G_ (%) = 0.68 × BM time (min) + 19.27 × NaOH concentration (%) + 13.71 (*R*^2^ = 0.85), *Y*_G_ = 6.35 × glucan content − 231.84 (*R*^2^ = 0.84), and *Y*_G_ =  − 14.22 × lignin content + 282.70 (*R*^2^ = 0.87).

**Conclusion:**

The combined wet alkaline mechanical pretreatment at room temperature had a boosting effect on the yield of enzymatic hydrolysis with short treatment time and less chemical consumption. The impact of the physical and chemical properties of corn stover pretreated with different BM times and/or different NaOH concentrations on the subsequent enzymatic hydrolysis was investigated, which would be beneficial to illustrate the effective mechanism of the mechanochemical pretreatment method.

**Supplementary Information:**

The online version contains supplementary material available at 10.1186/s13068-022-02130-0.

## Background

With the extensive development and application of fuel ethanol worldwide, lignocellulosic biomass, such as straw, has become a raw material source for fuel ethanol production owing to its advantages of large output and availability [1–5]. However, lignocellulosic biomass has a complex microstructure and chemical composition. A dense macromolecular network composed of cellulose, hemicellulose, and lignin in the cell wall of lignocellulosic biomass improves its recalcitrance of cellulose to chemical reagents and enzymes, seriously hindering ethanol production [6]. Therefore, pretreatment of lignocellulosic biomass is generally required to reduce its natural resistance and increase the ethanol production efficiency [7].

Using sodium hydroxide (NaOH) solution, alkaline pretreatment is a common and efficient chemical pretreatment method for lignocellulose. This process improves the accessibility of cellulose to enzymes by cleaving ester and ether bonds in lignin and hemicellulose through degreasing and saponification and removing lignin and some hemicellulose in plant cell walls [8–10]. The alkaline pretreatment process does not require expensive and complicated equipment, but long reaction times or high reaction temperatures are often needed to achieve the desired efficiency in the subsequent enzymatic hydrolysis [11]. For example, Li et al. mixed corn stover with 7 wt% and 10 wt% NaOH solutions at a mass ratio of 1:10, followed by grinding for 30 min at 140 and 160 °C, obtaining the maximum glucose yield from enzymatic hydrolysis after pretreatment using 10 wt% NaOH at 160 ℃ [12].

Ball milling pretreatment is an environmentally friendly physical pretreatment method that can disrupt the dense and complex physical structure of plant cell walls, reduce the size of biomass particles, destroy the crystalline structure of cellulose, and increase the degree of exposure to cellulose through mechanical force, which results in increased enzymatic hydrolysis efficiency [13–15]. Ji et al. reported that the glucose yield from enzymatic hydrolysis of rice straw increased after BM for 20 min owing to the average particle size being at the cellular scale (< 30–50 μm), destruction of the straw cell wall structure, and reduced crystallinity [16]. Although the ball milling process is environmentally friendly, pollution-free, and cannot change the original chemical composition of biomass, its energy consumption is high [15].

Recently, to utilize the advantages of alkaline and mechanical pretreatments while avoiding the problems of single pretreatment, mechanochemical pretreatment has attracted research attention [17–19]. Barakat et al. mixed wheat straw with NaOH solution and ammonia (5% w/w) in a 5:1 (w/v) ratio at room temperature for 5 h, and then the mixtures were ball-milled after drying in an oven at 105 °C [20]. Chuetor et al. mixed bagasse with NaOH (5% w/w) at a ratio of 1:2 or 1:5 (w/v) for 3 h, followed by centrifugal grinding treatment after drying (maintaining moisture at 8–10%) in 60 °C oven [21]. The above combined mechanochemical treatments can enhance enzymatic hydrolysis efficiency, but the long treatment time and drying step are not favorable for industrial processes.

Therefore, to further reduce treatment times, reduce the dosage of chemical reagents and avoid energy consumption in the drying process, a combined wet alkaline mechanical pretreatment at room temperature was proposed in this study. Generally speaking, the disadvantage of alkaline pretreatment is that the pretreatment time is too long. This study provided a pretreatment method with short time and less chemical consumption, which is innovative. The relationships of the microstructure and chemical composition of corn stover treated with different BM times and/or different concentrations of NaOH with the corresponding glucose yield from enzymatic hydrolysis were analyzed. The composition change of liquid fraction was explored with a view to provide experimental data to elucidate the effective mechanism of the mechanochemical pretreatment method.

## Results

**Fig. 1:**
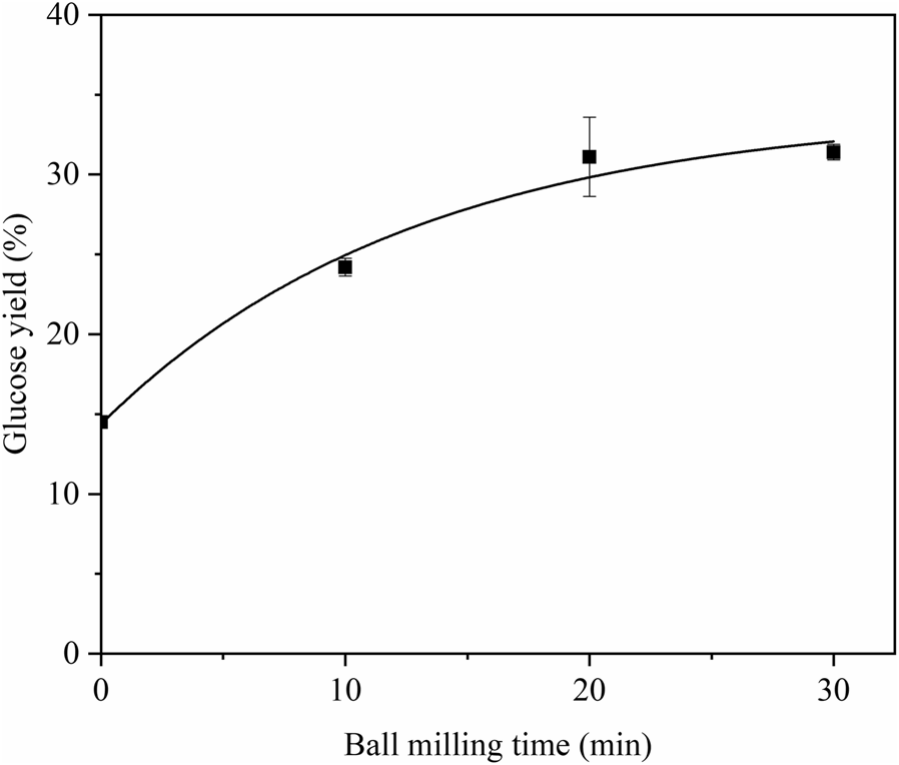
Glucose yield of corn stover treated with ball milling pretreatment

**Fig. 2:**
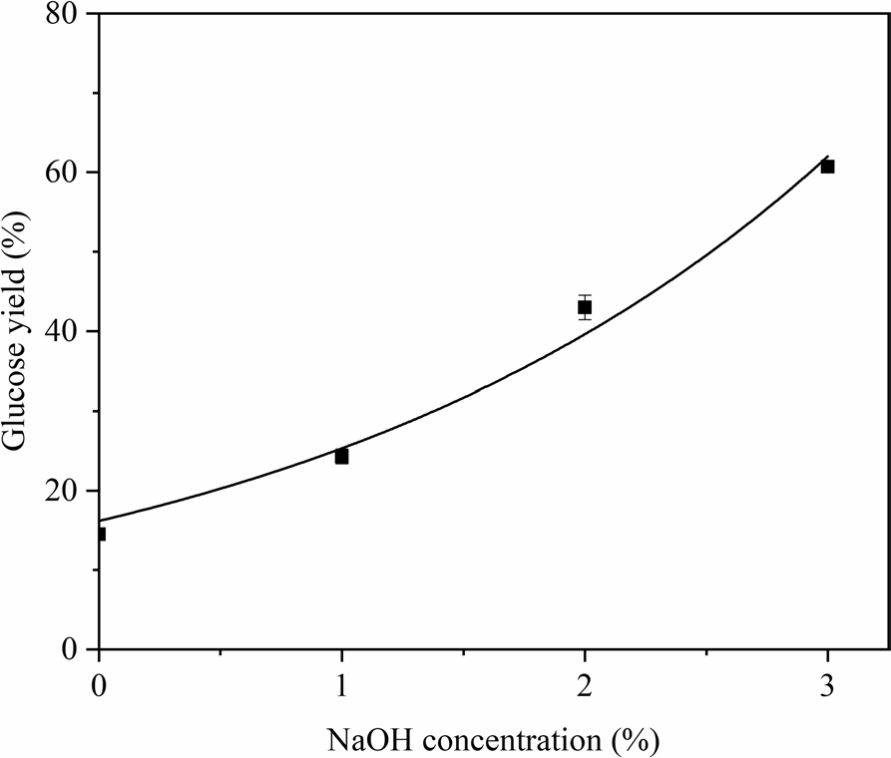
Glucose yield of corn stover treated with NaOH pretreatment

The sugar yields of the control treatment (WBM0-NaOH0%) were 14.5% of glucose yield and 4.7% of xylose yield. For samples treated only by ball milling (WBMx-NaOH0%), the yield of glucose and xylose increased with increasing ball milling time and reached a maximum of 31.2% glucose yield and 12.3% xylose yield at 20 min in Fig. 1. For corn stover only treated with NaOH (WBM0-NaOHy), the glucose yield and xylose yield significantly increased with increased NaOH concentration. At a NaOH concentration of 3%, the glucose yield was 60.7%, and the xylose yield was 42.6% in Fig. 2. The quantitative relationship between glucose yield and ball milling time, as well as glucose yield and NaOH concentration, can be found in Additional file 1. By comparison, the effect of NaOH treatment on the enhancement in enzymatic hydrolysis was higher than that of ball milling.

**Fig. 3:**
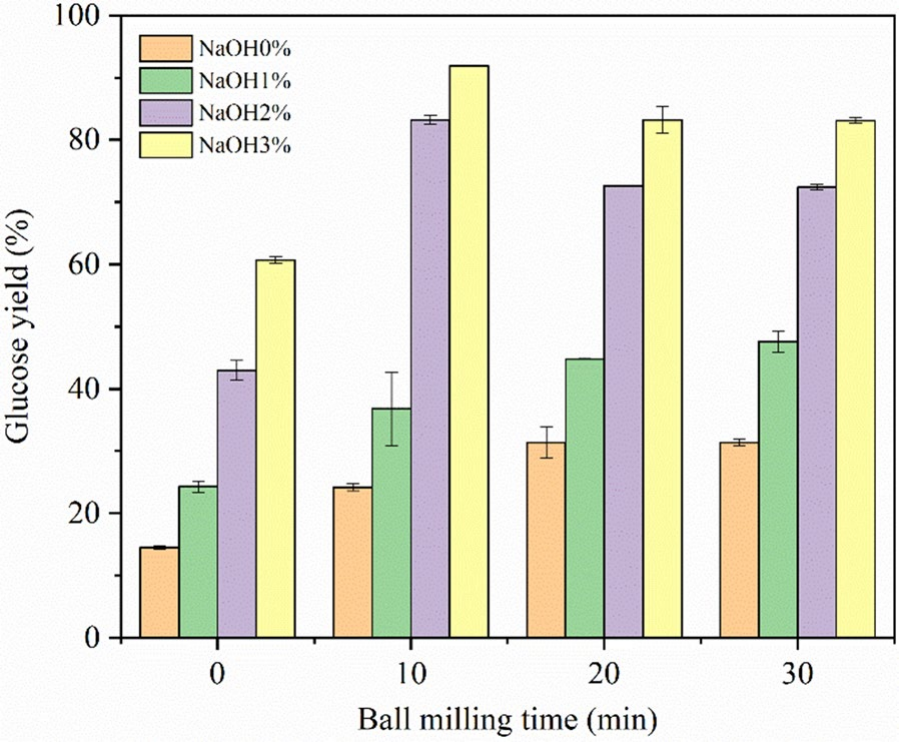
Glucose yield of corn stover treated with different pretreatments

**Fig. 4:**
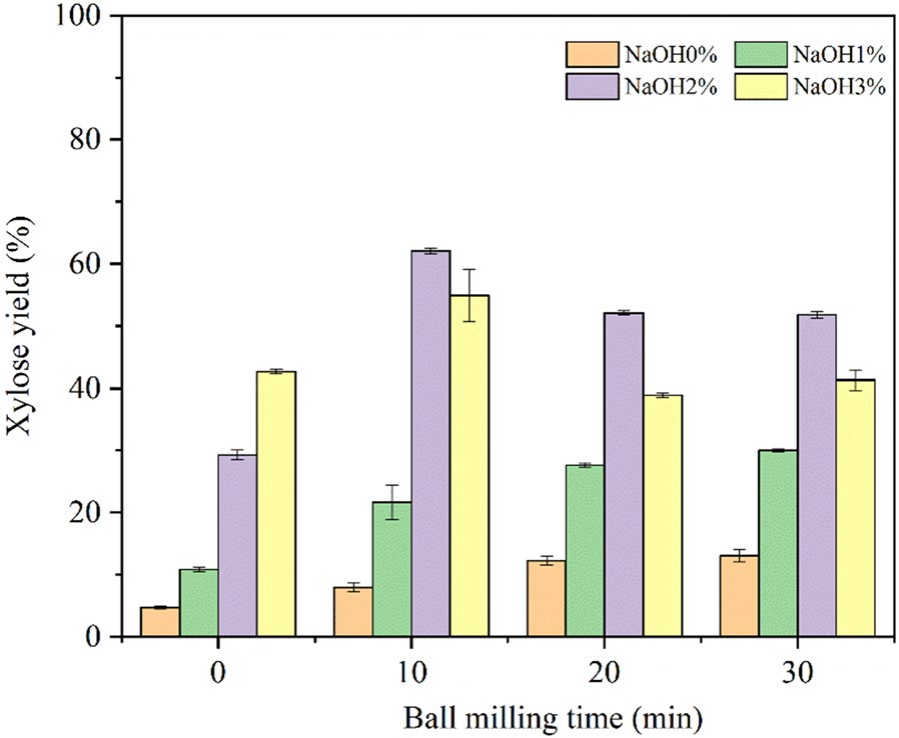
Xylose yield of corn stover treated with different pretreatments

**Fig. 5:**
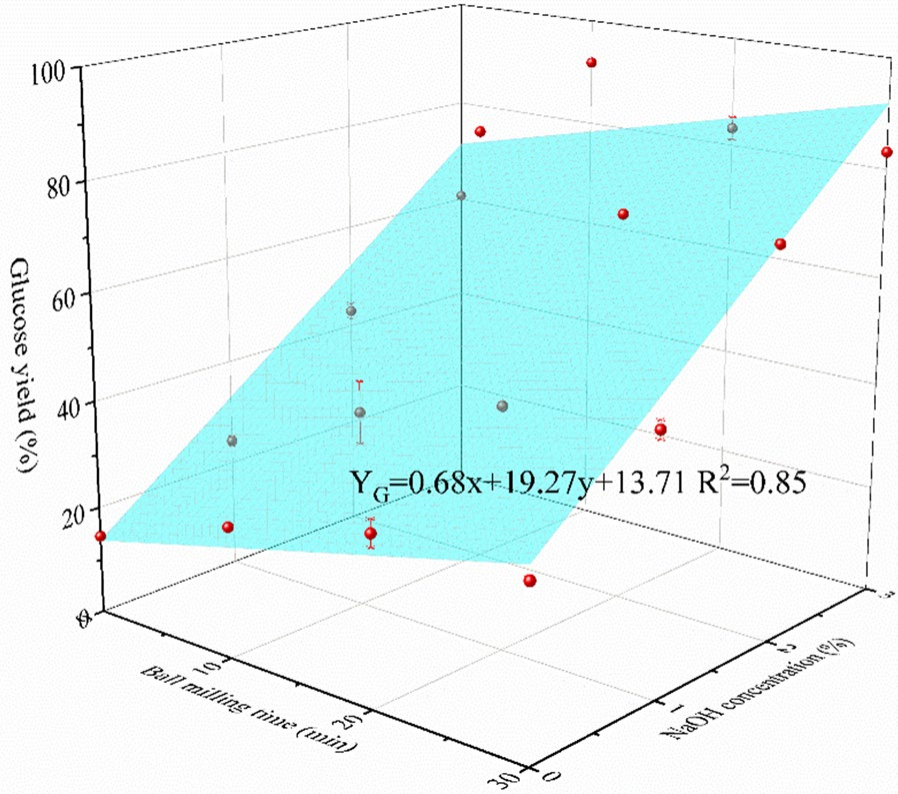
Glucose yield as a function of BM time and NaOH concentration

As can be seen from Fig. 3, with no NaOH to facilitate BM treatment, the glucose yield kept increasing with prolonged BM time. The maximum glucose yield without NaOH was only 31.4%. When 1% NaOH was added, the glucose yield (WBM30-NaOH1%) increased a little to 47.6%. The boost effect was significantly increased with 2% and 3% NaOH, resulting in 83.2% and 91.9% glucose yield, respectively. Besides, it only took 10 min to reach the maximum yield with 2% and 3% NaOH loadings. And the highest xylose yield of 62.1% could be obtained from WBM10-NaOH2% (Fig. 4). Therefore, compared with a single mechanical or chemical pretreatment method, combined wet alkaline mechanical pretreatment had a multiplicative effect on the glucose yield efficiency, which greatly enhanced the enzymatic hydrolysis from corn stover. Further binary analysis (Fig. 5) showed that the relationship of ball milling time and NaOH concentration with the glucose yield could be fitted with the following plane function: *Y*_G_ = 0.68x + 19.27y + 13.71 (*R*^2^ = 0.85).

## Discussion

**Fig. 6:**
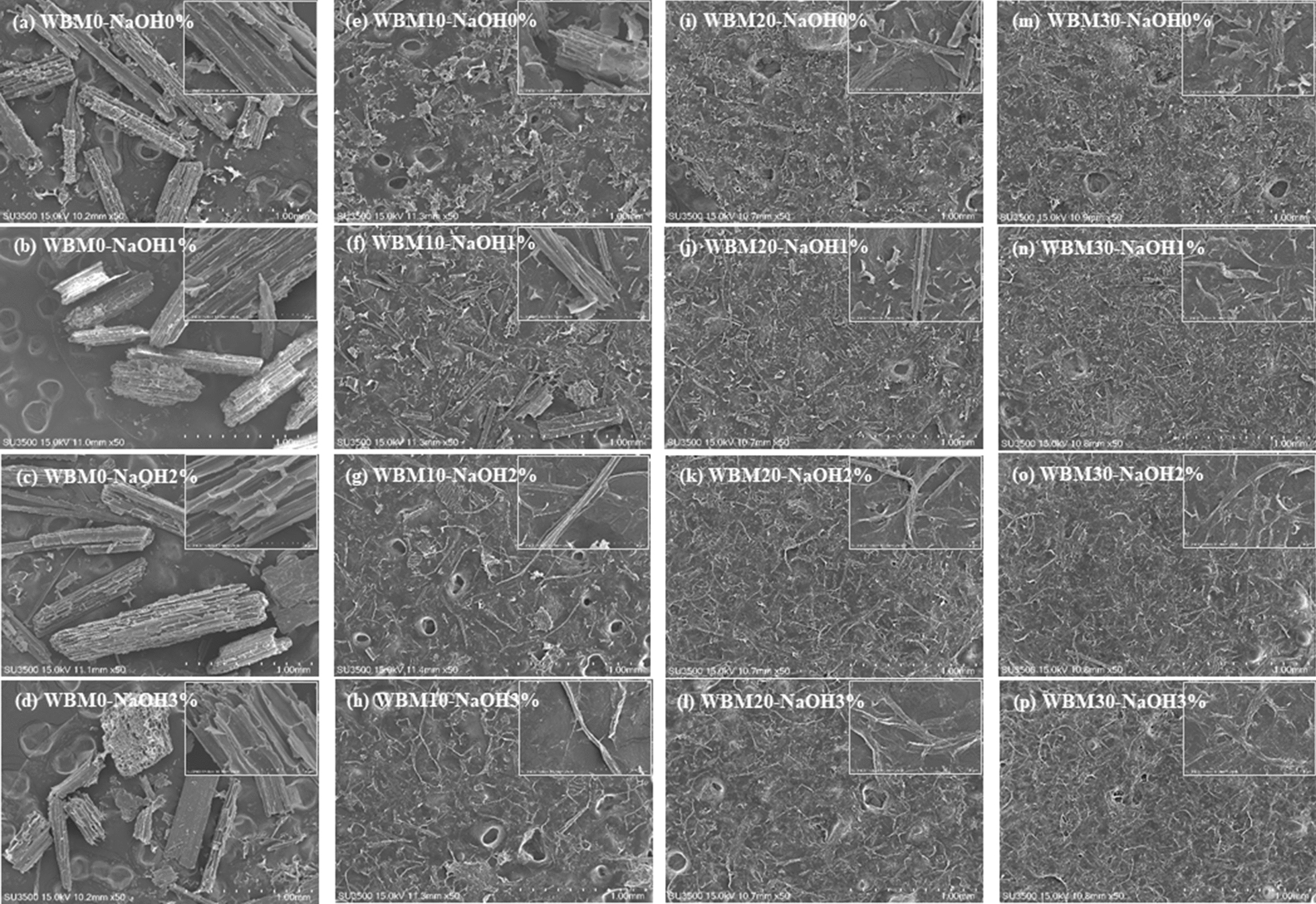
SEM images of corn stover samples from different pretreatments

According to scanning electron micrographs of corn stover from different pretreatments (Fig. 6), the particle size of samples was reduced after ball milling for 10 min, but the morphology and size of the particles did not change much with increased milling time.

**Fig. 7:**
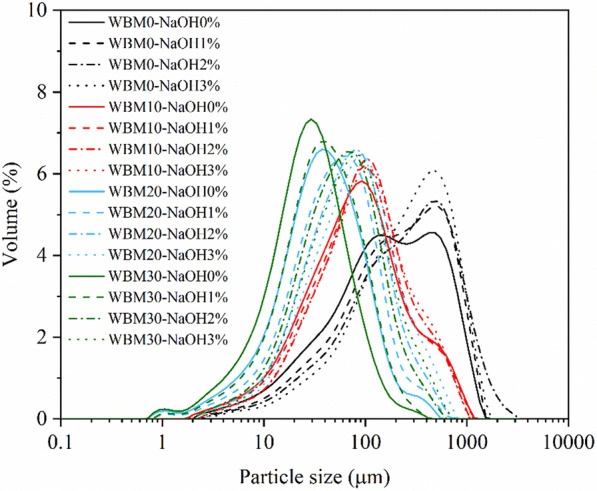
Particle size distribution curve of corn stover from different pretreatments

**Fig. 8:**
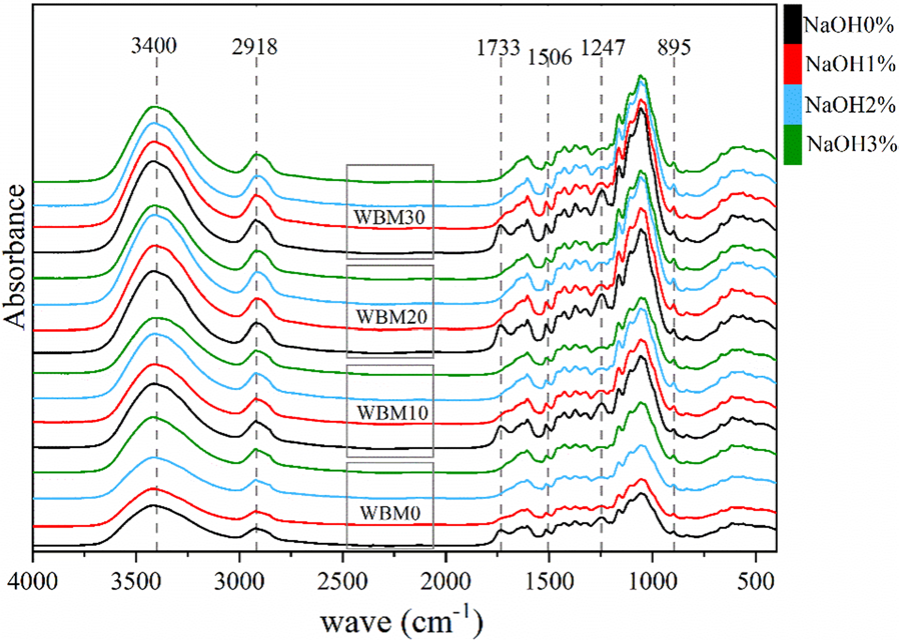
Infrared spectra of corn stover from different pretreatments

According to the results in Fig. 7 and Table 1, the average particle size (*D*_50_) of corn stover without mechanochemical treatment (WBM0-NaOH0) was 182.8 ± 8.2 μm, which was at the tissue scale (100–500 μm) [22]. For corn stover subjected to wet ball milling treatment, the *D*_50_ was reduced from 81.5 ± 1.2 μm at 10 min to 27.1 ± 0.2 μm at 30 min, which was at the cellular scale. The FT-IR curves of different samples did not show discernable changes, indicating that the wet ball milling treatment did not alter the functional groups of corn stover, which was consistent with previous reports (Fig. 8) [23]. The plant tissue structure and hydrogen bonds between crystalline cellulose were destroyed by mechanical forces, resulting in decreased crystallinity [15, 22]. Although BM treatment could not change the composition of samples, i.e., the glucan and xylan contents, as shown in Table 1, the cellulose exposure was increased owing to the increase in specific surface area, which was beneficial to improving the enzymatic hydrolysis efficiency to some extent [24–26]. For NaOH pretreatment, as shown in Table 1, the crystallinity of corn stover did not change much with increased NaOH concentration due to the overall NaOH loading being low [25, 26]. However, the D_50_ value increased (*p* < 0.01) owing to the swelling effect of NaOH solution on corn stover [21]. The absorption peaks at 1733 cm^−1^ attributed to C=O stretching and 1247 cm^−1^ correspond to the C–O stretching were disappeared, indicating that the ester bonds in hemicellulose and lignin were cleaved effectively by NaOH treatment (Fig. 8) [5, 22, 27]. The removal of lignin and xylose from the solids by NaOH resulted in a significant increase in the proportion of cellulose and a significant decrease in the lignin content (*p* < 0.01), while the xylan content decreased only slightly. Compared with the contents of the liquid fraction, it could be seen that the xylose content increased 127.8% from 15.9 mg/g (WBM0-NaOH 0%) to 36.2 mg/g (WBM0-NaOH 3%). The arabinose and acetic acid contents also showed a similar pattern of change, which indicated that the increase in NaOH concentration made more hemicellulose dissolved, resulting in no discernable increase of content in the solid [9]. Due to the nature of alkaline pretreatment, neither glucose nor xylose monomer will be released during the pretreatment [28, 29]. The sugars in the prehydrolysates were mainly in the form of oligomers, which represents up to 58.5% of glucose, and 91.2% of xylose (data not shown). According to previous research, lignin could cause non-productive binding with cellulase and limit the accessibility of xylan to enzymes [30–32]. Therefore, the glucose and xylose yield increased with the lignin content decreasing by NaOH pretreatment, which was consistent with the correlation analysis results in Table 2.

**Table 1:** Microstructural parameters and main chemical composition changes of corn stover from different pretreatments

Sample	*D*_50_ (µm)	Crystallinity (%)	Solid			Liquid					Solid yield (%)
			Glucan (%)	Xylan (%)	Lignin (%)	Glucose^1^ (mg/g^2^)	Xylose (mg/g)	Arabinose (mg/g)	Acetic acid (mg/g)	lignin (mg/g)	
WBM0-NaOH0%	182.8 ± 8.2^j^	43.8 ± 3.0^ef^	40.5 ± 0.3^ab^	22.1 ± 0.1^bcd^	19.4 ± 0.4^d^	46.5 ± 0.1^d^	15.9 ± 0.2^a^	2.1 ± 0.0^a^	1.1 ± 0.0^a^	7.8 ± 0.0^a^	91.1
WBM0-NaOH1%	226.6 ± 10.9^k^	42.9 ± 1.1^e^	42.4 ± 0.1^bcd^	23.0 ± 0.2^de^	17.8 ± 0.4^cd^	49.6 ± 0.2^g^	19.6 ± 0.1b^a^	3.4 ± 0.0^b^	25.4 ± 0.2^e^	11.9 ± 0.0^e^	80.0
WBM0-NaOH2%	250.2 ± 13.1^ l^	47.1 ± 0.2^f^	44.2 ± 0.3^edf^	23.2 ± 0.2^de^	16.7 ± 0.6^c^	51.8 ± 0.0^h^	25.1 ± 0.1^de^	5.8 ± 0.0^h^	33.3 ± 0.0^j^	15.9 ± 0.0^i^	73.6
WBM0-NaOH3%	283.8 ± 7.6^m^	43.6 ± 1.8^ef^	45.5 ± 1.2^fgh^	22.9 ± 0.6^de^	14.4 ± 0.6^a^	54.7 ± 0.2^j^	36.2 ± 0.3^f^	8.1 ± 0.1^i^	34.1 ± 0.0^ l^	17.2 ± 0.0^j^	74.5
WBM10-NaOH0%	81.5 ± 1.2^ fg^	41.5 ± 0.7^de^	39.5 ± 1.3^a^	21.4 ± 0.6^abc^	18.1 ± 0.7^ cd^	46.7 ± 0.1^ef^	21.9 ± 0.1^Bb^	3.4 ± 0.0^b^	1.7 ± 0.0^b^	9.3 ± 0.0^c^	93.2
WBM10-NaOH1%	86.5 ± 0.7^gh^	40.5 ± 0.9^cde^	42.7 ± 0.2^abcd^	23.5 ± 0.2^ef^	16.5 ± 0.9^bc^	41.9 ± 0.1^c^	20.5 ± 0.0^b^	4.8 ± 0.0^f^	29.6 ± 0.2^g^	15.3 ± 0.0^h^	79.3
WBM10-NaOH2%	94.0 ± 0.8^hi^	41.2 ± 0.4^de^	46.6 ± 0.8^ghi^	22.4 ± 0.6^cde^	14.8 ± 0.1^ab^	47.0 ± 0.0^f^	52.4 ± 0.1^ g^	16.7 ± 0.0^j^	31.4 ± 0.0^i^	24.5 ± 0.0^ m^	72.4
WBM10-NaOH3%	96.1 ± 0.6^i^	42.7 ± 1.4^e^	48.1 ± 0.2^ijk^	21.1 ± 0.2^ab^	14.3 ± 0.1^a^	60.2 ± 0.1^ m^	98.8 ± 0.1^i^	24.1 ± 0.0^ l^	35.7 ± 0.0^ m^	26.0 ± 0.0^o^	65.4
WBM20-NaOH0%	37.3 ± 0.2^b^	36.6 ± 0.6^ab^	39.9 ± 1.2^a^	22.2 ± 0.7^bcd^	17.0 ± 0.4^c^	56.5 ± 0.2^ k^	24.2 ± 0.2^d^	3.7 ± 0.0^c^	2.2 ± 0.1^c^	9.1 ± 0.0^b^	92.0
WBM20-NaOH1%	50.3 ± 0.2^c^	38.4 ± 1.0^bcd^	44.7 ± 0^efg^	24.4 ± 0.4^f^	17.4 ± 0.4^c^	46.2 ± 0.0^d^	22.6 ± 0.1^Ad^	5.0 ± 0.0^g^	28.1 ± 0.1^f^	15.0 ± 0.0^g^	80.8
WBM20-NaOH2%	65.3 ± 0.3^de^	37.4 ± 1.5^abc^	47.7 ± 0.9^hij^	23.0 ± 0.2^de^	14.8 ± 0.5^ab^	49.8 ± 0.1^g^	56.0 ± 0.0^h^	17.7 ± 0.0^k^	31.0 ± 0.1^h^	25.5 ± 0.0^n^	67.5
WBM20-NaOH3%	77.2 ± 0.3^f^	35.2 ± 0.7^ab^	50.1 ± 0.2^kl^	21.0 ± 0.1^ab^	14.5 ± 0.3^a^	58.5 ± 0.0^l^	102.2 ± 0.3^j^	24.1 ± 0.0^l^	33.7 ± 0.2^k^	22.6 ± 0.0^ l^	63.2
WBM30-NaOH0%	27.1 ± 0.2^a^	33.6 ± 0.4^a^	41.6 ± 0.9^abc^	22.9 ± 0.3^de^	17.7 ± 1.0^c^	40.4 ± 0.1^b^	25.6 ± 0.2^e^	3.9 ± 0.0^d^	1.3 ± 0.0^a^	11.7 ± 0.0^d^	91.3
WBM30-NaOH1%	37.7 ± 0.1^b^	36.1 ± 1.1^ab^	43.2 ± 0.6^cde^	23.6 ± 0.4^ef^	16.5 ± 0.9^bc^	37.6 ± 0.2^a^	19.8 ± 0.0^b^	4.5 ± 0.0^e^	23.6 ± 0.2^d^	13.2 ± 0.0^f^	83.2
WBM30-NaOH2%	56.8 ± 0.1^cd^	34.0 ± 0.1^a^	49.6 ± 0.7^jkl^	23.5 ± 0.4^ef^	14.4 ± 0.3^a^	54.0 ± 0.0^i^	56.1 ± 0.3^h^	17.7 ± 0.1^k^	34.3 ± 0.1^l^	20.2 ± 0.0^k^	62.0
WBM30-NaOH3%	67.4 ± 0.4^e^	35.4 ± 0.5^ab^	50.7 ± 0.6^l^	20.5 ± 0.1^a^	14.3 ± 0.0^a^	61.7 ± 0.2^n^	103.3 ± 1.3^k^	25.3 ± 0.2^m^	35.6 ± 0.1^m^	22.6 ± 0.0^l^	62.1

^1^The total sugar content of glucose, xylose and arabinose includes monosaccharide and oligosaccharide

^2^mg/g corn stover before pretreatment

Different lowercase letters in the same column indicate significant differences (*p* < 0.01)

**Table 2:** Pearson correlation analysis

	NaOH concentration (%)	WBM (min)	*D*_50_ (µm)	Crystallinity (%)	Glucan (%)	Xylan (%)	Lignin (%)	Glucose yield (%)	Xylose yield (%)
NaOH concentration (%)	1	0	0.260	0.43	0.893^**^	− 0.310	− 0.897^**^	0.879^**^	0.819^**^
WBM (min)		1	− 0.837^**^	− 0.945^**^	0.338	− 0.003	− 0.282	0.310	0.204
*D*_50_ (µm)			1	0.807^**^	0.090	0.070	0.057	− 0.167	− 0.065
Crystallinity (%)				1	− 0.304	0.029	0.258	− 0.252	− 0.124
Glucan (%)					1	− 0.266	− 0.884^**^	0.923^**^	0.846^**^
Xylan (%)						1	0.333	− 0.350	− 0.090
Lignin (%)							1	− 0.944^**^	− 0.905^**^
Glucose yield (%)								1	0.938^**^
Xylose yield (%)									1

**Indicates extremely significant correlation between parameters (*p* < 0.01), *Indicates significant correlation between parameters (*p* < 0.05)

**Fig. 9:**
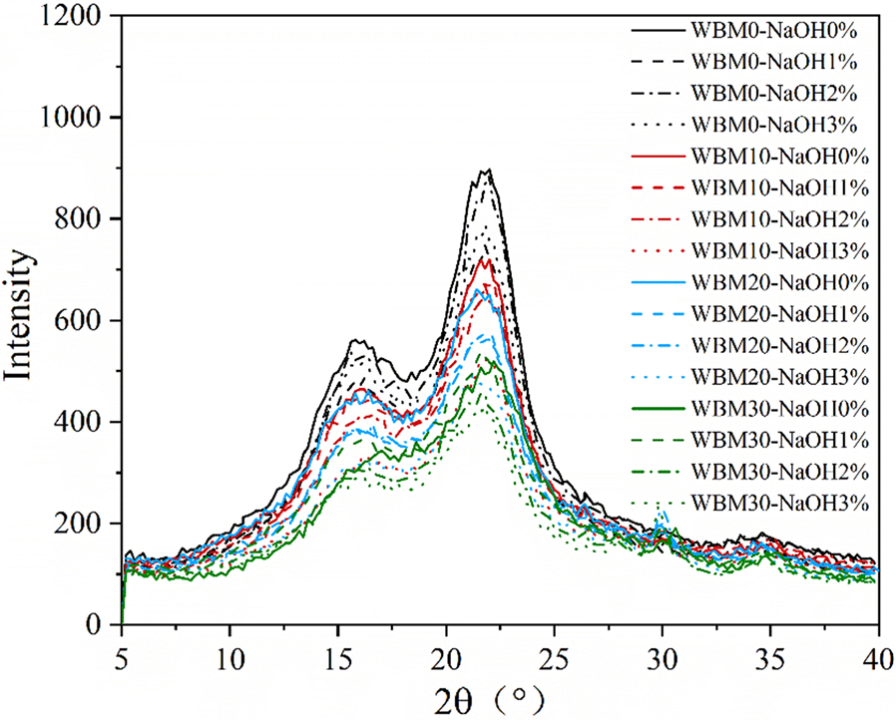
X-ray diffraction pattern of corn stover from different pretreatments

There was no furfural or HFM formation during the combined wet alkaline mechanical pretreatment. For the combined pretreatment, a discernable decrease trend in particle size was observed with prolonged BM time. Although the particle size of samples treated with NaOH was larger than the ones without NaOH treatment, the BM could still significantly reduce the particle size, e.g., 30-min of BM could reduce the particle size of samples treated with 3% NaOH from 283.8 to 67.4 μm (Table 1). And the cellulose characteristic diffraction peak intensity weakened with increased ball milling time (Fig. 9). The peaks around 1247 cm^−1^ and 1506 cm^−1^ correspond to the stretching or bending groups around the aromatic rings of lignin weakened compared with the control. The absorption peaks at 3400 cm^−1^, 2900 cm^−1^, 1200–1000 cm^−1^, and 895 cm^−1^ are related to the glucan content; the increase in these peaks indicated the increase of glucan content, which was consistent with the results in Table 1 [14]. Furthermore, the data in Table 1 showed that samples subjected to mechanochemical pretreatment had decreased size and crystallinity, with a trend similar to that observed for samples subjected to single ball milling pretreatment. The changes in chemical characteristics caused by single NaOH treatment, including the increased glucan content and lignin removal, were further enhanced by mechanochemical pretreatment. For example, the glucan content of WBM0-NaOH0% was 40.5%, which increased to 45.5% treated with 3% NaOH and 50.7% (86.6% retained) treated with 3% NaOH for 30 min BM time. Furthermore, the decrease in crystallinity and increase in average particle size caused by the swelling effect of NaOH contributed to promoting the diffusion of cellulase molecules and the accessibility of glucan to the enzyme, which resulted in a much greater effect on the glucose yield, as shown in Fig. 1 [25].

As shown in Table 2, the ball milling time had a negative effect on the *D*_50_ value and crystallinity (*p* < 0.01). The NaOH concentration had a positive effect on the glucan content, glucose yield, and xylose yield, while it had a negative effect on the lignin content (*p* < 0.01), consistent with the above analysis results. It is worth noting that there was no discernable correlation between xylose yield and xylan content. May the removal of lignin increase the xylose yield with slight changes of xylan content in the solid, leading to an irrelevant relationship between xylose yield and xylan content.

**Fig. 10:**
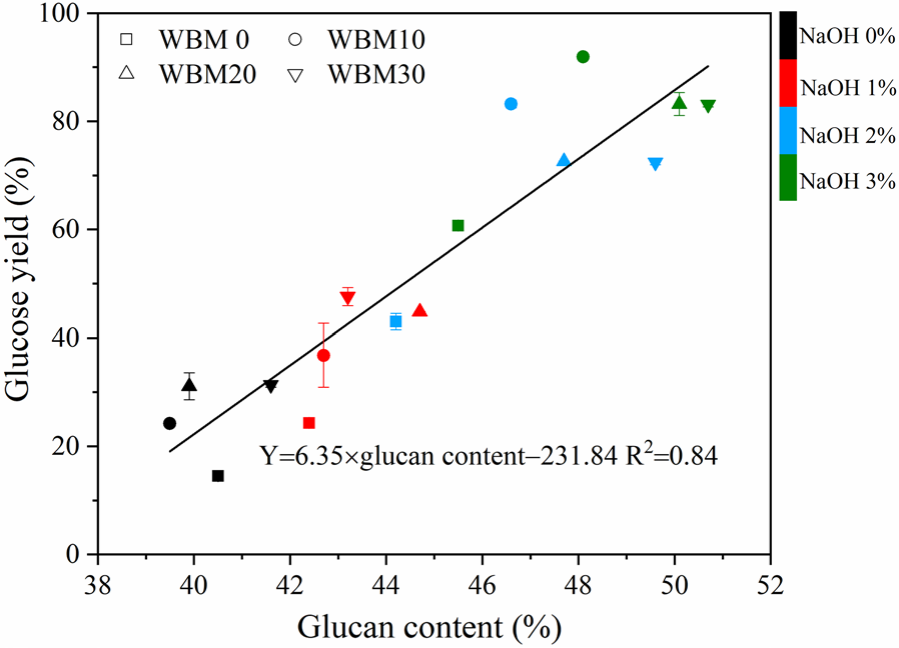
Relationship of glucose yield from corn stover with glucan content

**Fig. 11:**
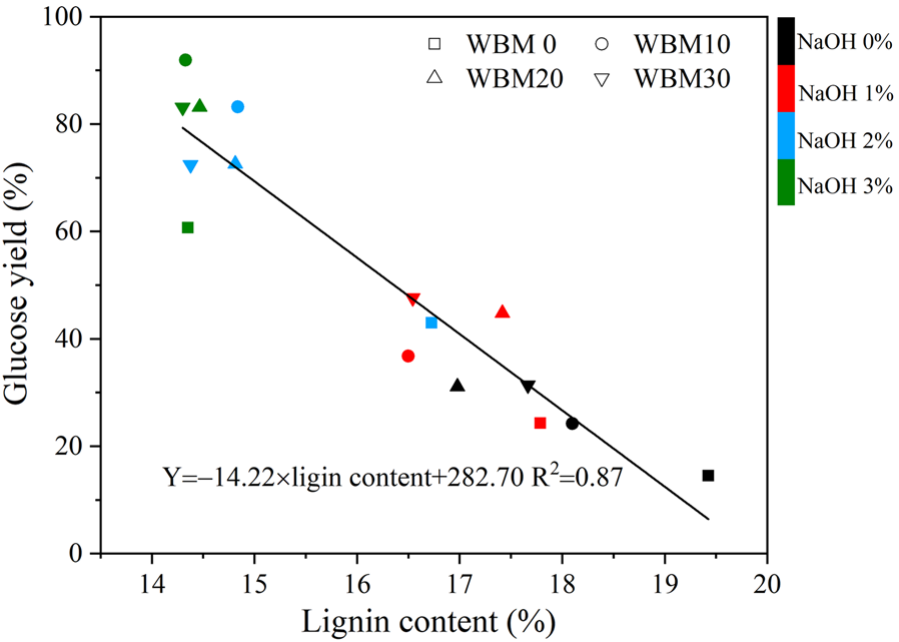
Relationship of glucose yield from corn stover with lignin content

The glucan content had a positive effect on the glucose yield (*p* < 0.01), while there was no relationship between glucose yield with *D*_50_ and crystallinity. For example, the glucan content reached a maximum after 10 min ball milling in combined alkaline mechanical pretreatment, and further changes in the chemical composition with an extended ball milling time were limited. It was similar to the changing trend in glucose yield, as shown in Fig. 1, which may explain the ball milling time and glucose yield showed no clear linear correlation in Table 2. The scatter plot of glucose yield and cellulose content is shown in Fig. 10, where the relationship was described as the following linear regression equation: *Y*_G_ = 6.35 × glucose content − 231.84 (*R*^2^ = 0.84). Li et al. found that the glucose yield of different samples treated with ball milling, alkaline hydrogen peroxide, and ammonia fiber expansion could be fitted with a univariate linear correlation with their glucan content [26]. It is consistent with the author’s results. Also, the glucan content of the substrate increases regardless of different pretreatments, and the glucose yield will increase accordingly within a certain range [26, 33]. The lignin content had a negative effect on the glucose yield (*P* < 0.01). The scatter plot of glucose yield, and lignin content is shown in Fig. 11, in which the relationship was described as the following linear regression equations: *Y*_G_ =  − 14.22 × lignin content + 282.70 (*R*^2^ = 0.87). Loustau-Cazalet et al. mentioned that the main reason for enhancing enzymatic hydrolysis was the removal of lignin and lignin–carbohydrate complexes rather than the change of cellulose crystallinity in a study of NaOH-VBM pretreatment [34]. Previously, Ishiguro et al. found a negative linear correlation between glucose yield and lignin content in obtained eucalyptus treated with hydrothermal–mechanical chemical [35]. Yang and Wyman et al. demonstrated that lignin removal facilitated the degradation of corn stover by cellulase [36]. However, Li et al. discovered an unclear correlation between glucose yield and lignin content when studying corn stover by different pretreatment, and Kumar et al. revealed that the reason why lignin hinders glucose yield might depend on its chemical properties rather than content [26, 37]. Combined with the previous data, it can be seen that the lignin content of WBM0-NaOH3% and WBM10-NaOH3% (44.4% lignin removal) is basically the same (14.4% and 14.3%, respectively), but the glucose yield of the latter is higher (60.7% and 91.9%, respectively). It may cause by changes in physical properties such as loosening of structure, reduction in particle size and crystallinity, and chemical properties such as increased glucan content through wet ball milling treatment. The fitted equation between glucose yield and lignin content has some limitations, which the wet ball milling treatment may cause. It reflects the enhanced effect of the wet ball milling on the NaOH treatment in the combined mechanochemical pretreatment, while there are limits to this enhancement, as shown by the fact that extending the ball milling time had little effect.

## Conclusions

A combined wet alkaline mechanical pretreatment of corn stover with short time and less chemical consumption at room temperature was proposed in this study, which significantly enhanced the subsequent enzymatic hydrolysis. A highest glucose yield of 91.9% was obtained after treatment with 3% NaOH and ball milling for 10 min, which was much higher than the yield from 10 min BM treatment alone (24.2%) and 3% NaOH treatment alone (60.7%). A major portion (86.6%) of glucan was retained at this optimal condition and 44% lignin was removed. The highest xylose yield of 62.1% was obtained with 2% NaOH and ball milling for 10 min. The quantitative relationships between glucose yield with NaOH concentration, ball milling time, glucan content, and lignin content were established. Compared with the BM time, the effect of NaOH concentration on the enhancement of yield was more obvious. Moreover, the component of liquid fraction after pretreatment as by-product was determined, and less harmful inhibitors were generated in this pretreatment process. The combined wet alkaline mechanical pretreatment in this study could become an effective pretreatment method in improving the efficiency of enzymatic hydrolysis.

## Materials and methods

### Corn stover and reagents

Corn stover collected from the Shangzhuang Experimental Station in Beijing, China, was dried naturally, chopped into small pieces (3–5 cm), and coarsely milled through a 1-mm sieve using an RT-34 milling machine (Hongquan Pharmaceutical Machinery Ltd., China), with the resulting milled corn stover denoted as CM. Its main components were 36.3% glucan, 18.4% xylan, 4.2% arabinan, and 16.9% lignin.

Sodium hydroxide (purity, ≥ 96.0%) was purchased from Beijing Chemical Plant and used without further purification.

### Mechanochemical treatments of corn stover

CM samples and NaOH solutions of different concentrations (0, 1, 2, and 3 wt%) were thoroughly mixed in a 1:6 (w/w) ratio (optimized from our pre-experiment) and were allowed to stand for a certain period (t_1_). An ultrafine vibration ball mill (CJM-SY-B; Qinhuangdao Taiji Ring Nano Ltd., Hebei, China) was then used for crushing the above mixture for different amounts of time *t*_2_(0, 10, 20, and 30 min) under the optimized conditions obtained from our preliminary tests, in which ZrO_2_ balls and CM were mixed in a volume ratio of 2:1, with the ZrO_2_ balls occupying 35% of the ball mill tank volume. Circulating cooling water was passed around the tank to maintain the temperature at around 25 °C during the milling process. All samples were in contact with NaOH solutions of different concentrations for 1 h (*t*_1_ + *t*_2_ = 1 h). The samples obtained were denoted as WBMx-NaOHy, where x was the milling time, and y was the NaOH concentration. The control treatment was as follows: CM samples were mixed thoroughly with deionized water for 1 h, without ball milling.

After the treatment, the whole slurry was neutralized by dilute hydrochloric acid before solid–liquid separation with a Buchner funnel. The liquid was collected and volume was recorded. A portion of the solids (washed) from treated corn stover was stored at 4 °C for subsequent enzymatic hydrolysis, and the remaining solid was dried in a vacuum freeze dryer to determine the lignocellulosic composition and crystallinity. The solid yield was calculated using Eq. (1) [38]:

1 $$\text{Solid yield} \left(\%\right)= \frac{{m}_{1}}{{m}_{2}}\times 100\%,$$

where *m*_*1*_ and *m*_*2*_ are the masses (g) of dry matter after and before treatment, respectively.

### Particle size distribution measurement

The particle size distribution of treated samples (diluted to 1 wt% of the original concentration for measurement) was obtained using a MASTERSIZER 3000 laser particle size analyzer (Malvern, UK) [39]. Particle sizes D_10_, D_50_, and D_90_, representing 10%, 50%, and 90% of the accumulated volume fraction, respectively, were determined using the obtained particle size distribution curves. Each sample was measured five times.

### Cellulose crystallinity (CrI) analysis

The cellulose crystallinity (*CrI*) of the obtained dried samples was measured using an XD3 series X-ray diffractometer (Puxi, Beijing) with Cu Ka radiation at 36 kV and 20 mA. The diffraction intensity was obtained in the 2θ range of 5–40° with a step size of 0.2° at a scanning speed of 2°/min. Each sample was measured in duplicate. The *CrI* was calculated according to Eq. (2) [40]:

2 $$CrI \left(\%\right)=\frac{{\mathrm{I}}_{\text{max}}-{I}_{\text{am}}}{{I}_{\text{am}}}\times 100\%,$$

where *I*_max_ is the maximum intensity of the diffraction peak at approximately 2θ = 22°, and *I*_am_ is the intensity of the amorphous background at approximately 2θ = 18°.

### Scanning electron microscopy (SEM) analysis

The surface morphologies of treated corn stover were observed using a Hitachi SU3500 electron microscope (Hitachi, Japan). Samples with a concentration of 0.1 wt% were dropped onto carbon tape, dried in an oven at 60 °C overnight, and sprayed with gold before subjecting to SEM observation.

### Fourier transform-infrared spectroscopy (FTIR) analysis

The functional groups within corn stover were detected with Fourier transform-infrared spectroscope (Spectrum 400; PerkinElmer; USA). Samples and spectroscopic KBr were thoroughly ground and mixed at a mass ratio of 1:100 in an agate mortar, then pressed using a tableting machine from a transparent sheet, which was placed onto the stage for scanning. The scanning wave number ranged from 400 cm^−1^ to 14,000 cm^−1^, the resolution was 4 cm^−1^, and the number of scans was 64. Each sample was measured in duplicates.

### Analysis of main chemical components

The cellulose, hemicellulose, and lignin contents in the solid were measured using the method of NREL-TP-510-42618 [41]. Each sample was measured in duplicates.

The total sugar content (monosaccharide and oligosaccharide) of glucose, xylose and arabinose, and byproduct contents such as furfural, 5-hydroxymethylfurfural (HFM), acetic acid in liquids were measured according to NREL-TP-510-42623 [42]. The acid-soluble lignin content was measured according to method NREL-TP-510-42618. Each sample was measured in duplicates.

### Enzymatic hydrolysis of corn stover

CellicCtec2 enzyme (Novozymes, Denmark) was used in the enzymatic hydrolysis of pretreated corn stover. It was purchased from Sigma Aldrich with an activity of 199.7 FPU/mL and protein number of 114.8 mg protein/mL. Pretreated samples (0.5 g dry basis) and citrate buffer (pH 4.8) were mixed in a 1:20 (w/v) ratio and were kept at 200 rpm and 50 °C for 72 h in a shaking incubator. Tetracycline hydrochloride (0.08 g/L) was added and the enzyme loading was 20 FPU/g solid, according to the method of NREL/TP-510-42623 [43]. Each sample was measured in duplicates. The glucose and xylose yield were calculated by Eq. (3) and (4), respectively [44]:

3 $$\text{Glucose yield}\left(\%\right)=\frac{\text{glucose released}*0.9}{\text{glucan in substrate}}\times 100\%,$$

4 $$\text{Xylose yield}\left(\%\right)=\frac{\text{xylose released}*0.88}{\text{xylan in substrate}}\times 100\%.$$

### HPLC analysis

The sugar contents were quantified by the HPLC (Hitachi, Japan) equipped with an Aminex HPX-87P column. Nano-pure water was used as a mobile phase running at 0.6 mL/min. The column temperature was 80 ℃, and the elution time was 40 min. Acetic acid, HMF, and furfural were quantified by the HPLC system equipped with an HPX-87H column. The mobile phase was composed of 5 mM of sulfuric acid running at 0.6 mL/min. The column temperature was kept at 55 °C, and the elution time was 50 min.

### Statistical analysis

The results of repeated experiments were expressed as means ± standard deviation. One-way analysis of variance was performed using Duncan’s test at the 99% level (*p* < 0.01) using SPSS 20.0 software. Data fitting was performed using Origin 2018 software.

## Supplementary Information

**Additional file 1.** The correlation between glucose yield and ball milling time or NaOH concentration.

## Data Availability

All data generated or analyzed during this study are included in this published article.
