# Supplementary material for: Effect of combined wet alkaline mechanical pretreatment on enzymatic hydrolysis of corn stover and its mechanism
Source: Biotechnol Biofuels Bioprod. 2022 Mar 17;15:31. doi: 10.1186/s13068-022-02130-0 (PMC8932242; doi:10.1186/s13068-022-02130-0)
Supplement: Supplementary file 1 — Additional file 1. The correlation between glucose yield and ball milling time or NaOH concentration. [file 13068_2022_2130_MOESM1_ESM.docx]

The relationship between glucose yield (Y_G_) and ball milling time (x) can be fitted as the following exponential function, Y_G_=33.97–19.61*0.93^x^ (R^2^=0.96).





Fig. S1 Glucose yield as a function of ball milling time.

The quantitative relationship between glucose yield and NaOH concentration (y) was represented by the following equation: Y_G_=16.19exp (0.45y) (R^2^ = 0.98).





Fig. S2 Glucose yield as a function of NaOH concentration.
